# Multimedia Image Data Analysis Based on KNN Algorithm

**DOI:** 10.1155/2022/7963603

**Published:** 2022-04-12

**Authors:** Runya Li, Shenglian Li

**Affiliations:** ^1^Research Institute of Finance, Hebei Finance University, Baoding, Hebei 071051, China; ^2^School of Management, Hebei Finance University, Baoding, Hebei Province 071051, China

## Abstract

In order to improve the authenticity of multispectral remote sensing image data analysis, the KNN algorithm and hyperspectral remote sensing technology are used to organically combine advanced multimedia technology with spectral technology to subdivide the spectrum. Different classification methods are used to classify CHRIS 0°, and the results are analyzed and compared: SVM classification accuracy is the highest 72 8448%, Kappa coefficient is 0.6770, and SVM is used to classify CHRIS images from five angles, and the results are compared and analyzed: the classification accuracy is from high to low, and the order is FZA = 0 > FZA = −36 > FZA = −55 > FZA = 36 > FZA = 55; SVM is used to classify the multiangle combined image, and the result is compared with the CHRIS 0° result: the overall classification accuracy of angle-combined image types is lower than that of single-angle images; the SVM is used to classify the band-combined image, and the result is compared with CHRIS 0°: the overall classification accuracy of the band combination image forest type is very low, and the effect is not as good as the combining multiangle image classification results. It is verified that if CHRIS multiangle hyper-spectral data are used for classification, the SVM method should be used to classify spectral remote sensing image data with the best effect.

## 1. Introduction

**Figure 1:**
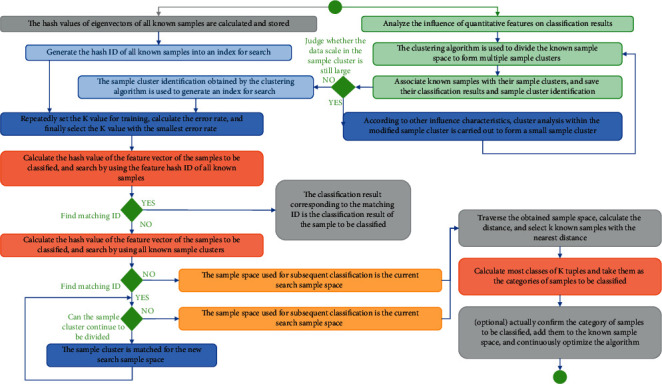
Flow chart of KNN algorithm.

Machine learning is a process of automatically or semiautomatically finding patterns from massive data. As shown in Figure 1, once a pattern is found, it is repetitive. Different interpreters can get the same results by applying this model to other similar data [1], which greatly improves work efficiency, and the credibility of the results is comparable. At present, the commonly used machine learning methods include decision tree, artificial neural network, KNN, support vector machine, and random forest. KNN classification algorithm is a typical nonparametric learning method. It has been widely used in many fields because of its simplicity of implementation and high classification accuracy. It has always been a hot issue in data mining, machine learning, and statistical pattern recognition research. The algorithm examines the K samples that are most similar to the sample to be classified and determines the category of the sample to be classified according to the category attributes of the K samples. The most similar K samples are determined by the distance between the sample to be classified and the training sample. In the K-nearest neighbor classifier, the choice of the K value is very important. If the choice of K value is too small, it will not fully reflect the characteristics of the sample to be classified. But, when the K value is selected too large, some samples that are not similar to the samples to be classified are also included, which leads to the reduction of the classification effect [2]. It also has some shortcomings: KNN is a lazy learning method, and it caches all training samples and does not establish a classification until the data need to be classified. When the training set size is large or the dimensionality is high, the classification efficiency will be lowered by 0.8. The traditional pixel-based classification method uses a pixel as a unit for image processing. When the image area is large, the classification efficiency will be seriously affected. Object-based image analysis uses objects as primitives instead of pixels for image classification. In addition to the advantages of making full use of the spectrum, texture, shape, size, and context of ground objects, it can greatly reduce the amount of image processing data and improve classification efficiency. Based on the eCognition 9.0 software, the paper uses the Landsat-8 fusion data in Zhongwei City, Ningxia, as the data source and uses the KNN classifier to perform pixel-based and object-based classification. It also compares the classification characteristics of images using the same training samples, verification samples, and feature data sets and explores the advantages of integrating multispectral remote sensing image classification based on object image analysis and the KNN algorithm [3].

## 2. Literature Review

Spectral characteristics are an important basis for remote sensing methods to detect the properties and shapes of various substances. Qin et al. found that matter under the interaction of electromagnetic waves, due to electronic transitions, atomic and molecular vibration and rotation, and other complex effects, spectral absorption and reflection characteristics that reflect the composition and structure of the matter will be formed at certain specific wavelength positions [4]. Further research by Arun and Govindan found that these diagnostically significant spectral features, such as absorption peak width, are generally only a few nanometers to tens of nanometers [5]. Ulhaq et al. found that especially in the red edge feature called the vitality indicator of plants, the displacement is generally only a few nanometers to tens of nanometers [6]. Li et al. believe that such fine spectral information is difficult to reflect in the resource satellite remote sensing data of hundreds of nanometers (nm) in the widely used bandwidth at present [7]. In other words, there are only a few discrete imaging bands in the visible light-near-infrared-shortwave infrared range, and the traditional remote sensing method with a spectral resolution of several hundred nanometers is difficult to meet the requirements of further in-depth and detailed study of material properties and composition. Xing and Li's need for in-depth study of material properties and even composition is increasing day by day [8]. In order to make up for the shortcomings of traditional remote sensing and expand the potential of remote sensing applications, people have been committed to the theoretical research and practical use of continuous narrow bands to obtain spectral information of ground objects for more than 20 years. This combination of spectral images with high spectral resolution in the continuous spectral range (visible light to near-infrared, short-wave infrared, and even thermal infrared), with tens or even hundreds of bands, has become an imaging spectroscopy technology. Bing and others believe that the continuous improvement of spectral resolution is another important trend in the development of remote sensing [9]. Du et al. found that remote sensing with a spectral resolution in the order of 10-1 is called multispectral, commonly known as conventional or traditional remote sensing. Such remote sensing sensors have only a few bands in the visible light and near-infrared spectral regions, such as the Landsat series of the American Earth resource satellite and the SPOT series of the French resource satellite. Remote sensing with a spectral resolution of 10-2A is called hyperspectral remote sensing. Because its spectral resolution is as high as nanometers in the visible light region, it often has the characteristics of multiple bands, and its spectral channels in the visible light to the near-infrared spectral region are as many as dozens to hundreds. With the further improvement of remote sensing spectral resolution, when it reaches 10-3A, remote sensing enters the ultraspectral stage. At this time, the spectral resolution is as high as 0.2–1 nm0 in the visible and near-infrared spectral regions [10]. At present, multispectral imaging and hyperspectral imaging have been realized. On the basis that multispectral remote sensing has been widely used, hyperspectral remote sensing has also entered the test and practical stage. Yesilbudak and others believe that the ultrahigh spectral resolution is currently mostly nonimaging methods. Hyperspectral remote sensing is an inevitable development that people expect to obtain more information through remote sensing methods, and it is one of the frontiers and hotspots of contemporary remote sensing [11]. Zhang et al. found that hyperspectral remote sensing has brought strong vitality to remote sensing applications with the rich spectral information carried by its high spectral resolution characteristics [12]. Li et al. found that, compared to wide-band multispectral remote sensing, hyperspectral remote sensing has brought the classification of ground objects from the classification of vegetation, roads, cultivated land, residential areas, and water bodies to the fine classification of different species of the same species. It brings the macroscopic understanding of vegetation to the microscopic realm of the study of plant biochemical components, showing a huge and attractive prospect [13].

## 3. Methods

The main idea of the KNN algorithm is to first calculate the distance or similarity between the sample to be classified and the training sample of a known category and find the K neighbors whose distance or similarity is closest to the sample to be classified; then, according to the category to which these neighbors belong, the type of sample data to be classified is determined. The specific steps of the algorithm are as follows:

(1) The training set *S* is searched and found the *k*-nearest neighbor samples closest to the object *d* to be classified. “Nearest Neighbors” use distance metrics, such as Euclidean distance. The Euclidean distance of two objects *X*_1_=(*x*_11_, *x*_12_,…, *x*_1*n*_) and *X*_2_=(*x*_21_, *x*_22_,…, *x*_2*n*_) is
  (1) $$\begin{array}{c} \text{dist}\left(X_{1},X_{2}\right)=\sqrt{\overset{n}{\underset{i=1}{\sum }}{\left(x_{1i}-x_{2i}\right)}^{2}}. \end{array}$$
The choice of distance measurement is very important, and other distances can also be used for measurement.
(2) The weight of *d* belonging to each class is calculated, and the weight calculation formula of *d* belonging to *C*_*j*_ is
  (2) $$\begin{array}{c} W\left(d,C_{i}\right)=\sum_{i=1}^{k}\text{sim}\left(d_{i},d\right)\ell \left(d_{i},C_{j}\right), \end{array}$$
where sim(*d*_*i*_, *d*) is the similarity between *d* and the *i*-th nearest neighbor object *d*_*j*_
(3) The weight of the class is compared, and the class to be classified is assigned to the class with the largest weight.

At present, hyperspectral remote sensing has been applied to forest mapping, forest resource survey, forest area monitoring, biochemistry, and physical investment estimation. However, most of the data that can be used are obtained by aerial imaging spectrometers. Spaceborne imaging spectrometers have been successfully developed, but they are in the experimental stage [14, 15]. As of November 2008, there are more than forty high-quality airborne instruments in the world, and only three satellite-borne hyperspectral sensors have been successfully launched. One is the Hyperion carried by the test satellite E0-1 launched by the United States on November 21, 2000. It is an Atlas measuring instrument used to measure the spectral characteristics of the target and to image the target; it is compatible with the multispectral imager. The main difference is high spectral resolution and many spectrum bands. The other two similar instruments are (1) FTHSI (Fourier transform hyperspectral imager), installed on the U.S. Air Force's MightySat-2.1 satellite, launched on July 19, 2000; (2) the high-resolution imaging spectrometer CHRIS carried on the small satellite PROBA launched by the ESA organization on October 22, 2001.  Table 1 shows the main performances of the stars currently in orbit in the hyperspectral range.

There are few spaceborne hyperspectral sensors, mainly because some technical problems are still difficult to solve on satellites. The difference between spaceborne and airborne is caused by the great difference in height. For the same ground resolution, the instantaneous field of view (IFOV) of the spaceborne instrument is tens to hundreds of times smaller, and the corresponding instrument sensitivity is tens of times higher. Several hundred times, this is one of the difficulties; because the difference in the radiation characteristics of the detection elements is more obvious in the case of the input signal, it is no easy task to obtain the detection elements with good uniformity. This is the second difficulty. Judging from the published images after the launch of the EO-1 satellite, the quality is good, but the quantitative evaluation of its geometry and radiation characteristics needs to be verified after verification [16, 17]. Based on the CHRIS multiangle hyperspectral remote sensing data and the understanding of the investigation of the test area, using multiangle hyperspectral remote sensing data, mainly centering on the subject of forest type classification, the following key work has been progressed. The specific content is as follows.

**Figure 2:**
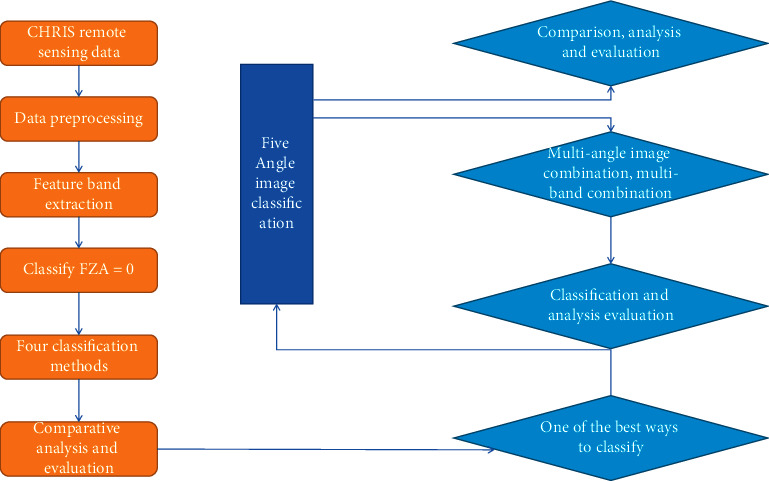
Technical flow chart.

1. Based on an in-depth understanding of the spectral characteristics of multiangle hyperspectral remote sensing data, a set of preprocessing procedures for CHRIS multiangle hyperspectral remote sensing data is summarized, including striping, atmospheric correction, orthorectification, and MNF transformation.
2. In order to explore the best classification method, FZA = 0 data are taken as an example, and different classification methods are used to classify image data, analyze, and compare the results and obtain a more ideal classification method.
3. Using the most accurate and ideal classification method, the five multiangle hyperspectral data of CHRIS were classified into forest types, and the results were compared and evaluated.
4. Based on the conclusions drawn from the above research, the methods of multiangle combination and band combination are explored to improve the classification accuracy of forest types, and various multiangle and band combination images are classified, analyzed, and evaluated. The technical route is shown in Figure 2.

CHRIS acquires visible light and near-infrared spectroscopy data in a push-broom method. CHRIS products are divided into two levels, Level 0 and Level 1, and LO is the original data, which is only used to produce L1 products. Users are using L1 products, and L1 products have only one data format, namely HDF (hierarchical data format) Hlxe. Its characteristics are shown in Table 2. CHRIS is a small satellite-borne remote sensing hyperspectral imager, including a telescope and an imaging spectrometer connected to a 770-column 576-row CCD multiple-column detection system. There are 5 imaging modes in CHRISL1 data: modes 3, 4, and 5 are mainly land imaging, mode 2 is water imaging, and mode 1 includes land and water imaging. The specific classification is as follows:

  MODE 1: All column widths, 62 spectrum bands, the spectrum range is 773∼1036 nm, and the ground resolution of the nadir point is 34 m
  MODE 2: Water band: all column widths, 18 spectral bands, and the ground resolution of the nadir point is 17 m
  MODE 3: Land band: all column widths, 18 spectral bands, and the ground resolution of the nadir point is 17 m
  MODE 4: Chlorophyll band settings: all column widths, 18 spectral bands, and the ground resolution of the nadir point is 17 m
  MODE 5: Land band: half column width, 37 spectral bands, and the ground resolution of the nadir point is 17 mlk41. The MODE4 vegetation remote sensing data of CHRIS are with the spectral range between 490nm–796nm. The characteristics of the CHRIS data used in the paper are shown in Table 2:

As can be seen from Table 3, the experimental data source CHRIS has a total of 18 bands, with a spatial resolution of 16 m, and simultaneously acquires hyperspectral data from five different angles (0, 36°, −36°, 55°, and −55°). The product has not been geometrically corrected.  Table 4 shows the center wavelength and spectral width of each band of CHRIS.

**Figure 3:**
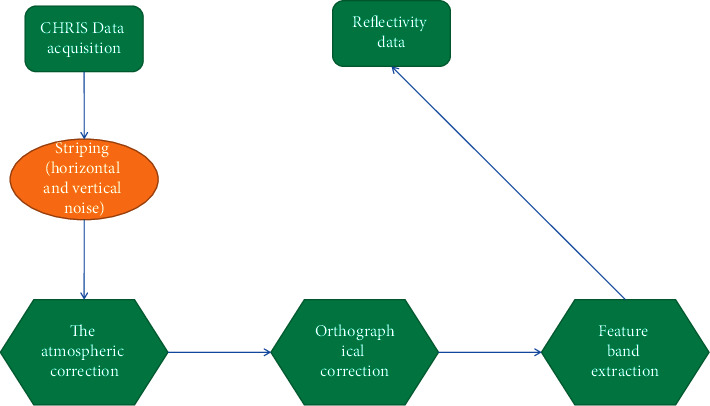
CHRIS data preprocessing process.

It can be seen from Table 4 that the CHRIS hyperspectral remote sensing data are used in this paper: band 1 is the blue band, and the spectral range is 484.4∼496 nm; band 2 is the green band, and the spectral range is 544.9∼557.8 nm; band 3∼15 is the red light band, and the spectral range is 631.2∼758.8 nm; the band 16∼18 is the near-infrared band (VNIR), and the spectral range is 777∼796 nm. Hyperspectral remote sensing technology organically combines advanced imaging technology and spectroscopy technology to subdivide the spectrum and obtain images of the Earth's surface with high spectral resolution on the order of nanometers (nm). It contains extremely rich information; that is, it contains rich spatial and spectral information. The imaging spectrometer receives the electromagnetic wave radiation (reflection and emission) from the ground target and records it in the form of an image gray scale through digital-to-analog conversion, vividly showing the spatial distribution characteristics of the surface material, the interconnection, and the occurrence of geographic processes, laws of development, and evolution. Multiple bands are the most significant feature of hyperspectral data. Conventional remote sensors only acquire images in a few discrete wavebands, and the waveband width is generally 100–200 nm, while the hyperspectral technology is to perform continuous spectral imaging of the target with a waveband width on the order of nm. In this way, while obtaining the spatial image of the ground surface, the continuous spectral information of the ground objects can also be obtained [18, 19]. If the wavelength is the horizontal axis and the gray value is the vertical axis, the gray value of each pixel point on the hyperspectral image in each channel can form a fine spectral curve, which is the so-called “map unification.” The ultra-multiband of hyperspectral data constitutes its unique ultra-multidimensional spectral space. Therefore, when analyzing and extracting information, what we are concerned about is the distribution characteristics of different types of ground objects in this n-dimensional spectral space. On the premise of preserving important information, how to project from high-dimensional data space to low-dimensional space, eliminate or reduce the correlation between data dimensions, reduce data dimensionality, reduce intraclass differences, and increase class spacing. Therefore, it is more convenient, fast, effective, and accurate to extract useful information [20, 21]. The CHRIS data preprocessing process is shown in Figure 3. The CHRIS/PROBA reflectance image is returned after preprocessing.

## 4. Results and Analysis

**Figure 4:**
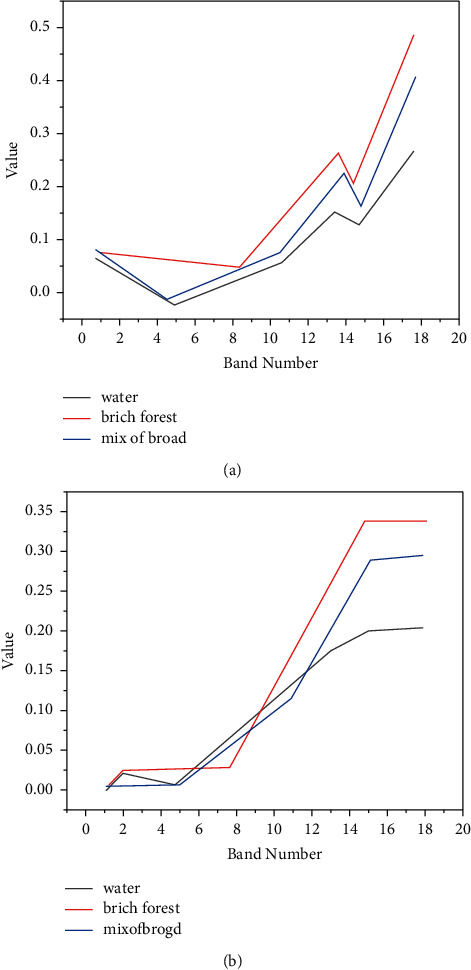
FZA = 0 comparison of spectrum curves before and after atmospheric correction. (a) Spectrum curve before correction. (b) Spectral curve after correction.

All kinds of radiant energy used by remote sensing (here mainly refers to solar short-wave radiation energy) interact with the Earth's atmosphere, such as scattering and absorption, which attenuates the energy and changes the spectral characteristics. The process to eliminate these atmospheric influences is called atmospheric correction [22, 23]. The process of remote sensing imaging is a complicated process. In the imaging process, the light signal passes through the atmosphere twice in the process from the sun to the sensor and cannot avoid being affected by the atmosphere. Due to the presence of the atmosphere, solar radiation is weakened by the absorption of atmospheric molecules and the scattering of aerosol particles. At the same time, part of the scattered signal enters the sensor directly or through the reflection of ground objects and is enhanced again. In actual processing, atmospheric influence reduces the contrast ratio of the image, reduces the readability of the image, and increases the difficulty of interpretation. At present, the most widely used atmospheric correction method is the radiation transmission model method with the best physical meaning and the highest accuracy. This method uses a model established by the principle of electromagnetic wave radiation transmission in the atmosphere to perform atmospheric correction on remote sensing images. The most widely used model is the 6S model, LOWTRAN model, MORTRAN model, ACTOR2/3 model. Among them, the 6S model is based on the theory of radiation transmission. The model has a wide range of applications and is not affected by the characteristics of the study area and the types of targets. Combining actual remote sensing images and existing meteorological conditions, considering that the CHRIS map is small, it can be considered that the aerosol is uniform within the map [24, 25]. Furthermore, the important atmospheric parameters on the day the CHRIS image was taken are obtained from the local meteorological department. Now, the 6S atmospheric physical radiation transmission model is selected as the correction tool for correcting the CHRIS image, and the acquired important atmospheric parameters are used as the input parameters of the 6S. Because the 6S parameter file needs to enter the solar zenith angle, azimuth angle, and observation zenith angle, azimuth angle, combined with CHRIS data, the solar azimuth angle needs to be calculated here. Assuming that the azimuth of the sun is medium, the calculation formula is

(3) $$\begin{array}{c} \cos\;\ell =\frac{\sin\;\ell -\sin\;\varphi \;\cos\;\alpha \theta }{\cos\;\varphi \;\sin\;\theta }, \end{array}$$

where *ℓ* is the declination of the sun, that is, the latitude of the direct sun point, which is a function of each day of the year and has nothing to do with the location of the observation point, *θ* is the sun zenith angle, and *φ* is the latitude of the observation target. The azimuth angle *φ* of the sun must be calculated by the formula. The output file can be obtained after running the program. After running the 6S model, the pixel value is a value between 0 and 100. You can divide by 100 to get the reflectance or multiply by 2.25 to get the gray value image as needed. From Figure 4, it is not difficult to find that the atmospheric correction effect is very obvious, and the spectral curve is more in line with the spectral characteristics of vegetation [26, 27]. After the image is corrected by the atmosphere, the ground reflectance data are obtained, which plays a role in improving the accuracy of remote sensing application research.

## 5. Conclusion

Hyperspectral remote sensing technology is the development frontier of remote sensing technology in the 21st century and one of the focuses of remote sensing in the world today. It mainly uses five different angles of image classification, and the experimental results show that FZA = 0 has the highest overall classification accuracy. However, it does not mean that the classification accuracy of each vegetation type is the highest. For example, the classification accuracy of water bodies is 53%, but the classification accuracy of water bodies in the classification results of FZA = 36 and FZA = −36 is higher than that of FZA = 0, which is 78% and 72%, respectively. If the classification accuracy of the water body is required to be high, the classification image with FZA = 0 should not be used, but the classification result image with FZA = 36 or FZA = −36 should be selected [28], the classification result image with FZA = −36 should be selected, and FZA = 0 to classify images cannot be used. It suggests that, in response to the needs of different types of high-precision features, the classification results of images from different angles are selected, and the five angles play a complementary role to some extent, verifying the importance of hyperspectral remote sensing technology.

## Figures and Tables

**Table 1:** Comparison of main performance of spaceborne hyperspectral sensors.

Parameter	MightySat/FTHSI	EO-1/Hyperion	PROBA/CHRIS
Launch time	2000.7.19	2000.11.21	2001.10.22
Spectral range	0.33 um∼1.04 um	0.5∼2.4 um	0.5∼1.05 um
Spatial accuracy	30m	30 m	I 8m
Width		7.5 km × 185 km	14 km × 14 km
Spectral coverage	Continuous	Continuous	Discontinuous
Spectral coverage	202	218	MI:62, M2- M4:18, M5:37
Repeat cycle/day		16	Irregular

**Table 2:** Characteristic description of CHRIS products.

Space adopted interval (m)	Nadir point 18
Image area (km ∗ km)	14 × 14
Number of images/piece	5 (different angle)
Image size (km)	13 × 13 (768 × 748 pixels)
Each image size (mbit)	131
Pixel format	BSQ
Spectral range (m)	400∼1050
Data unit	microWatts/mm/m 2/str
Number of spectral bands	18 bands with a spatial resolution of 17 m, and 62 bands with a spatial resolution of 34 m
Spectral resolution	1.3 nm@410 nm to 12 nm@1050 nm
Signal-to-noise ratio	200

**Table 3:** Characteristic description of CHRIS level1 products.

Sensor type	CHRIS
Get time	August 4, 2008 (02: 01)
Number of images/piece	5 (different angle)
Number of spectral bands	18 bands
Spatial resolution	6 m
Pixel format	BSQ
Data unit	microWatts/nm/m ∗ 2/str
Japanese standard latitude and longitude	127.79, 42.04 degree
Platform height	657 m
Observe the azimuth	185.28 degree
Observe the zenith angle	3.43 degree
Image size	766 ∗ 768
Spectral range	490∼800

**Table 4:** The center wavelength and spectral width of each band of CHRIS multiangle hyperspectral data.

Band number	Center wavelength	Spectral width	Band number	Center wavelength	Spectral width
1	491.1	11.5	10	712.4	6.3
2	552.2	12.7	11	718.6	6.3
3	634.2	14.2	12	734.8	13.3
4	667.9	10.6	13	744.8	6.8
5	68.9	11.5	14	751.8	7
6	689.4	5.9	15	758.7	7.1
7	697.2	5.5	16	776.8	14.8
8	701.2	6.1	17	788.2	7.5
9	705.2	6.3	18	796.1	7.6

## Data Availability

The data used to support the findings of this study are available from the corresponding author upon request.
